# Recombinant human TIM-3 ectodomain expressed in bacteria and recovered from inclusion bodies as a stable and active molecule

**DOI:** 10.3389/fbioe.2023.1227212

**Published:** 2023-07-31

**Authors:** G. C. Lima, R. M. Chura-Chambi, L. Morganti, V. J. Silva, M. P. Cabral-Piccin, V. Rocha, T. S. Medina, R. N. Ramos, D. Luz

**Affiliations:** ^1^ Laboratory of Bacteriology, Butantan Institute, São Paulo, Brazil; ^2^ Biotechnology Center, Institute of Energy and Nuclear Research—CNEN/SP, São Paulo, Brazil; ^3^ Laboratory of Medical Investigation in Pathogenesis and Directed Therapy in Onco-Immuno-Hematology, Department of Hematology and Cell Therapy, Clinical Hospital, Faculty of Medicine, University of São Paulo, São Paulo, Brazil; ^4^ International Research Center, A. C. Camargo Cancer Center, São Paulo, Brazil; ^5^ D’OR Institute of Research and Education, São Paulo, Brazil

**Keywords:** TIM-3, inclusion bodies, bacteria expression, active recombinant protein, membrane protein

## Abstract

**Introduction:** Microbial systems, such as *Escherichia coli*, as host recombinant expression is the most versatile and the cheapest system for protein production, however, several obstacles still remain, such as recovery of soluble and functional proteins from inclusion bodies, elimination of lipopolysaccharides (LPS) contamination, incomplete synthesis, degradation by proteases, and the lack of post-translational modifications, which becomes even more complex when comes to membrane proteins, because they are difficult not only to produce but also to keep in solution in its active state. T-cell Immunoglobulin and Mucin domain 3 (TIM-3) is a type I transmembrane protein that is predominantly expressed on the surface of T lymphocytes, natural killer (NK) cells, dendritic cells, and macrophages, playing a role as a negative immune checkpoint receptor. TIM-3 comprises a single ectodomain for interaction with immune system soluble and cellular components, a transmembrane domain, and a cytoplasmic tail, responsible for the binding of signaling and scaffolding molecules. TIM-3 pathway holds potential as a therapeutic target for immunotherapy against tumors, autoimmunity, chronic virus infections, and various malignancies, however, many aspects of the biology of this receptor are still incompletely understood, especially regarding its ligands.

**Methods:** Here we overcome, for the first time, the challenge of the production of active immune checkpoint protein recovered from bacterial cytoplasmic inclusion bodies, being able to obtain an active, and non-glycosylated TIM-3 ectodomain (TIM-3-ECD), which can be used as a tool to better understand the interactions and roles of this immune checkpoint. The TIM-3 refolding was obtained by the association of high pressure and alkaline pH.

**Results:** The purified TIM-3-ECD showed the correct secondary structure and was recognized from anti-TIM-3 structural-dependent antibodies likewise commercial TIM-3-ECD was produced by a mammal cells system. Furthermore, immunofluorescence showed the ability of TIM-3-ECD to bind to the surface of lung cancer A549 cells and to provide an additional boost for the expression of the lymphocyte activation marker CD69 in anti-CD3/CD28 activated human PBMC.

**Discussion:** Taken together these results validated a methodology able to obtain active checkpoint proteins from bacterial inclusion bodies, which will be helpful to further investigate the interactions of this and others not yet explored immune checkpoints.

## Introduction

The most commonly used organism for heterologous protein production, especially when it does not require post-translational modification, is the microbial system using the gram-negative bacterium *Escherichia coli* (*E. coli*). This organism is one of the most well-known and established models for biological processes so it is no surprise that most prokaryotic membrane protein structures found in the PDB have been obtained after the production of the corresponding protein in *E. coli* (Terpe, 2006; Hattab et al., 2014). However, even though *E. coli* is probably the most versatile and cheapest host for protein production, several obstacles remain: inclusion bodies formation, LPS contamination, incomplete synthesis, degradation by proteases, and the lack of post-translational modifications (Hattab et al., 2014).

T-cell Immunoglobulin and Mucin domain 3 (TIM-3), a type I transmembrane protein, is an immune checkpoint that belongs to the T-cell immunoglobulin mucin (Tim) family and was first described as a Th1-specific cell-surface marker, representing the first Tim family member to be associated with immune regulation (Monney et al., 2002). The TIM-3 structure architecture contains an extracellular region with a single membrane distal immunoglobulin variable (IgV) domain containing six invariant cysteines and a varying length membrane-proximal mucin domain, which are considered the ectodomain portion of the TIM-3 protein. The ectodomain interacts with immune system soluble and cellular components. Moreover, there is a single transmembrane domain and a cytoplasmic tail. The cytoplasmic domains consist of 78 amino acids responsible for the binding of signaling and scaffolding molecules through a tyrosine-based signaling motif (Cao et al., 2007). The TIM-3 native ectodomain binds to TIM-3’s cognate ligands and is primarily expressed on activated T and myeloid cells.

The engagement of this immune checkpoint by its ligands inhibits T-cell proliferation and cytokine production (Anderson, 2012). Therefore, the TIM-3 pathway holds potential as a therapeutic target for immunotherapy against tumors, autoimmunity, chronic virus infections, and various malignancies (Anderson and Anderson, 2006; Anderson, 2012; Attanasio and Wherry, 2016). Even though TIM-3, following its similar immune checkpoint proteins such as CTLA-4 and PD-1, gained interest as a target for immunotherapy, many aspects of its biology are not completely understood, especially when it comes to its ligands (De Sousa Linhares et al., 2020). Studies diverge regarding which interactions trigger the signaling pathway of TIM-3’s negative regulation role on immune cells (De Sousa Linhares et al., 2020). In this context, it is very important to characterize the possible TIM-3 interactions to be able to study its relevance in the immune context more deeply.

Obtaining soluble ectodomains of human membrane proteins is a challenging task, especially when using bacterial expression systems, mainly because these proteins may have complex structures, including multiple disulfide bonds, that are difficult to obtain in bacteria (Berkmen, 2012; Cruz and Kayser, 2019; Bhatwa et al., 2021). Moreover, bacterial expression systems may not always produce soluble proteins and may require the use of denaturants or harsh purification methods, which can potentially alter the protein’s structure, stability, and function (Williams et al., 1982; Burgess, 2009; Singh et al., 2012). In some cases, this can lead to the loss of important protein-protein interactions or even aggregate formation, which are not suitable for downstream applications (Dill and Shortle, 1991; Panda, 2003).

Therefore, obtaining human membrane soluble ectodomain proteins from bacterial expression systems often requires a combination of expression conditions optimization, such as alternative methods of inclusion bodies solubilization (Khan et al., 1998; Singh et al., 2015). Utilizing a mild solubilization process that avoids the complete unfolding of native-like protein structures is beneficial due to the demonstrated presence of native-like secondary structures and potential activity in inclusion body aggregates (Przybycien et al., 1994; García-Fruitós et al., 2005; Jevsevar et al., 2005; Ventura and Villaverde, 2006; Chura-Chambi et al., 2018; Chura-Chambi et al., 2022; Morganti and Chura-Chambi, 2023). Here, we demonstrate for the first time the refolding of TIM-3 from inclusion bodies by mild solubilization, using the association of high pressure and alkaline pH and the obtainment of a stable and active full ectodomain region of TIM-3 (TIM-3-ECD). This bacterial production strategy can be extended to obtain other complex cell surface receptors, such as immune checkpoints and their ligands, in a cheaper and faster manner. Furthermore, the purified TIM-3-ECD molecule will be used to further investigate non-dependent N-linked glycosylation interactions in the mediation of T cell regulation as well as to generate specific antibodies toward non-glycosylated epitopes.

## Materials and methods

### Cell culture

A549 epithelial cells (type II alveolar lung epithelium cells) were cultured in a 1:1 volume mixture of DMEM High Glucose (Gibco, United States) and Ham’s F12 (Gibco, United States) supplemented with 10% (v/v) fetal calf serum (Gibco, United States of). HEK-293 epithelial cells (embrionary kidney cells) were cultured in DMEM High Glucose (Gibco, United States) supplemented with 10% (v/v) fetal calf serum (Gibco, United States). PBMC were cultured in RPMI medium supplemented with 10 mM HEPES, 0.1 mM non-essential amino acids, 1 mM sodium pyruvate, 0.05 mM 2-mercaptoethanol, and 10% (v/v) fetal bovine serum (Gibco, United States). Cells were cultured at 37°C with 5% CO_2_.

#### TIM-3-ECD construct

The human TIM-3 ectodomain (NCBI Reference Sequence: NP_116171.3, 22aa-202aa) was cloned into the expression vector pET24a+, in-frame with an ATG initiation codon and a poly-histidine tail at C-terminal coding sequence. The synthesized sequence had codons optimized for expression in *Escherichia coli* (ref www.kazusa.or.jp/codon). Vector pET24a + contains a polyhistidine tail (6xHis) in the N-terminal portion, in-frame with the cloned gene to facilitate the purification process. The construct pET24a-TIM-3 was synthesized by the company GeneOne (Rio de Janeiro, Brazil).

#### Expression of recombinant TIM-3-ECD

Expression vector pET24a-TIM-3 was chemically transformed into a competent bacterial expression strain *E. coli* BL21 (DE3) and plated on LB-agar medium (50 μg/mL kanamycin), followed by incubation at 37°C for 18 h. A single colony was inoculated into 3 mL of Luria Bertani broth (LB) containing 50 μg/mL of kanamycin and incubated at 37 °C under 200 rpm shaking for 16 h. Inoculum was added at a 1:100 volume ratio in the LB medium (50 μg/mL kanamycin) into 2 L flasks and incubated at 37 °C under 200 rpm shaking until an optical absorbance density of 0.4–0.6 at 600 nm was attained. Expression induction occurred by adding 1 mM isopropyl-β-D-thiogalactopyranoside (IPTG) to the culture medium and incubating at 37 °C under 200 rpm shaking for 18 h. Bacterial cells were harvested at 6,800 x *g*, at 4 °C for 20 min, and stored at −20 °C for at least 24 h.

Cells were solubilized at a sample:buffer volume ratio of 20:1 in lysis buffer (50 mM NaH_2_PO_4_, 300 mM NaCl, 40 mM Imidazole, 200 μg/mL Lysozyme, 0.05% Triton-100 (v/v), 10 mM MgCl_2_, 100 Mm Phenylmethylsulfonyl fluoride (PMSF), and 100 mM Benzamidine, pH 8.0) using a T-25D disperser (IKA, Germany), followed by incubation at 4 °C under gentle agitation for 60 min. The sample was maintained on ice and submitted to a second lysis step by sonication using a HD2070 sonicator (Bandelin, Germany). Three cycles of 10 min (0.9 s on/1 s off) were performed, followed by centrifugation at 15,600 x *g*, at 4 °C for 60 min. Soluble and insoluble fractions were maintained separately.

### Solubilization of inclusion bodies

The insoluble fraction was washed in buffer A (100 mM Tris-HCl, 5 mM EDTA, 0.1% Sodium Deoxycholate (v/v), 1 mM PMSF, and 100 mM Benzamidine, pH 8.0) using a sample volume:buffer ratio of 20:1 followed by 2 min (0.9s on/0.1s off) of sonication using a HD2070 sonicator (Bandelin, Germany). The sample was centrifuged at 15,600 x *g*, at 4°C for 30 min, the supernatant was discarded, and the pellet was suspended in an equal volume of wash buffer A, repeating the sonication and centrifugation step. The pellet was solubilized in buffer B (50 mM Tris-HCl, 1 mM EDTA, and 1 mM PMSF, pH 8.0) at a culture volume:buffer ratio of 40:1 and the rounds of sonication were repeated. The sample was kept at −20°C.

The solubilization of inclusion bodies was performed at high hydrostatic pressure, as previously described (Morganti and Chura-Chambi, 2023). Briefly, the solubilized material was diluted in solubilization buffer (50 mM CAPS, 1 mM EDTA, 400 mM Arginine, and 20 mM Dithiothreitol, pH 10.0), packed in double vacuum sealed plastic bags, placed in a high-pressure vessel (R4-6-40, High Pressure Equipment, United States), and pressurized for 90 min at 2.4 kbar using a suitable high-pressure pump (PS-50, High Pressure Equipment, United States) that injected the transmission fluid (oil) in the vessel. After decompression, the sample was removed from the plastic bags and centrifuged at 15,600 x *g*, at 4 °C, for 15 min to remove insoluble aggregates. The sample supernatant was dialyzed at 4 °C for 18 h on a SnakeSkin™ Dialysis Tubing 10k MWCO dialysis membrane (Thermo Fisher Scientific, United States) with 100 volumes of 50 mM Tris-buffer without NaCl. After dialysis, the sample was quantified on a NanoDrop spectrophotometer (Thermo Fisher Scientific, United States of America) and analyzed on SDS-PAGE. All the following experiments were performed with recombinant TIM-3 diluted with 50 mM Tris-Buffer.

#### Immunodetection

After solubilization of inclusion bodies under mild denaturing conditions, immunodetection was performed to confirm the presence of histidine-tailed proteins. Protein fractions were analyzed by SDS-PAGE and transferred to an Amersham™ Protran™ 0.45 μm nitrocellulose membrane (GE, United States) using the Trans-Blot SD semi-dry transfer cell system (BioRad, United States) with transfer buffer (ethanol 20% (v/v), Tris-glycine 10% (v/v)). The membrane was incubated at 4 °C under gentle agitation for 16 h with blocking buffer (PB1% − PBS containing 1% of bovine serum albumin - BSA). The membrane was washed three times under gentle agitation for 5 min with PT buffer (PBS containing 0.05% of Tween-20).

Murine anti-HisTag primary antibody (Merck, Germany) was added at a 1:2,500 dilution in blocking buffer to the membrane in incubation under gentle agitation for 1 h. The membrane was washed three times for 5 min under gentle agitation with PT buffer and then the membrane was incubated with peroxidase-conjugated mouse anti-IgG secondary antibody (Merck, Germany) at a dilution of 1:10,000 in blocking buffer under gentle agitation for 1 h. The washing step was repeated and the reaction was developed by incubation with DABI (10 mg/mL 3,3′-Diaminobenzidine, 50 mM Tris-HCl 5, 150 mM NaCl, and 0.08% H_2_O_2_ (v/v), pH 7.6) under gentle rotation for 10 min and the reaction stopped with distilled water.

#### circular dichroism

The TIM-3-ECD secondary structure was analyzed by circular dichroism (CD). The CD spectra of samples, quantified in a NanoDrop spectrophotometer (Thermo Fisher Scientific, United States), were analyzed in the wavelength range between 190 and 260 nm using a 0.1 mm thick quartz cuvette in a JASCO J-810 spectropolarimeter (Jasco Corporation, Japan). After subtraction of noise coming from the sample buffer (Tris 25 mM), the CD data were converted to unit degree x cm^2^ x dmol^−1^ of mean residue ellipticity (θ). The results were analyzed on the BeStSel online server available at: www.bestsel.elte.hu/index.php.

#### Indirect enzyme-linked immunosorbent assay (ELISA)

A 96-well MaxiSorp Nunc plate (Thermo Fisher Scientific, United States) was coated with recombinant TIM-3-ECD or commercial TIM-3-ECD (His-tagged, Elabscience, United States) Log2 serial dilutions in PBS, starting at a concentration of 10 μg/mL and incubated at 4 °C for 18 h. The plate was washed three times with PT buffer followed by a blocking step via the addition of 200 μL/well of PB 1% buffer and incubated at 37°C for 2 h. The wash step was repeated and 100 μL/well of murine anti-TIM-3 primary antibody and cloneTI.H3 (GenScript, United States) diluted to 1:1,000 in PB1% was added followed by incubation at 37°C for 60 min. Next, 100 μL/well of rabbit antibody conjugated murine anti-IgG clone A9004 (Sigma-Aldrich, Germany) diluted to 1:10,000 in PB1% was added and the plate incubated at 37°C for 45 min. The assay was developed by incubating an OptEIA™ solution prepared following the manufacturer’s instructions (BD Biosciences, United States) under gentle agitation for 15 min. The reaction was stopped by stopping the addition of 1 M H_2_SO_4_ solution and reading the absorbance in a spectrophotometer at a wavelength of 450 nm.

#### Peripheral blood mononuclear cells (PBMC) stimulation assay with TIM-3-ECD

Aliquots of PBMC were obtained by Ficoll gradient from fresh blood samples of volunteers after written informed consent was obtained, in accordance with the Commission on Human Beings and the Ethics and Research Committee of the Clinical Hospital of São Paulo (process number CAAE: 52712521.0.000.0068). Cells were collected on the day of the experiment and washed twice with PBS buffer. The cells (1 × 10⁶ cells/tube) were incubated in 1 mL of RPMI medium supplemented with 10 mM HEPES, 0.1 mM non-essential amino acids, 1 mM sodium pyruvate, 0.05 mM 2-mercaptoethanol, and 10% (v/v) fetal bovine serum containing 50 μg/mL of recombinant TIM-3-ECD for 24 h. Control groups did not contain ectodomain addition to the culture medium.

After incubation with recombinant TIM-3-ECD, cultured PBMCs were stimulated with 5 × 10⁵ nanospheres adsorbed with anti-CD3 and anti-CD28 antibodies (Thermo Fisher Scientific, United States) at 37°C in a 5% CO_2_ environment for 12 h. Control groups did not contain the addition of mitogen agents to the culture medium. After incubation with mitogen agents, cells were transferred to cytometry tubes and incubated with monensin (Thermo Fisher Scientific, United States) to block Golgi complex activity. Cultures were then washed with PBS after centrifugation at 400 *g*, for 5 min and solubilized with 100 μL of conjugated antibody mixture diluted in PB 0.5% (anti-CD3 (Thermo Fisher Scientific clone HIT3a) Percy5 (2 μL/mL); anti-CD8 (Thermo Fisher Scientific RPA-T8) FITC (2 μL/mL); anti-CD69 (Thermo Fisher Scientific clone FN50); and APC (2 μL/mL), and incubated at 4°C for 20 min. Labeled cells were washed by centrifugation at 400 *g*, for 5 min with PB 0.5% and incubated on ice until analysis.

Flow cytometry analysis was performed on DxFlex equipment (Beckman-Coulter) and cytometry data were analyzed using FlowJo software (BD Biosciences).

#### Immunofluorescence of TIM-3-ECD

Immunofluorescence was performed using the lung cancer A549 cell line as the positive control because they express immune checkpoint receptors on their surface and the HEK293 cell line as the negative control because it is not a tumor cell. In total, 1 × 10^5^ cells were plated in sterile 24-well plates on circular glass slides to allow cell adhesion and spreading until reaching confluency. Recombinant TIM-3-ECD (50 μg/mL) was then added to the culture medium and incubated at 37 °C for 1 h. After incubation, cells were washed twice with 1 mL of serum-free culture medium and incubated with 1 mL of chilled 4% paraformaldehyde at 4 °C for 48 h for fixation. Next, cells were washed twice with 1 mL of PBS 2% glycine buffer and blocked with 1 mL of PB1% at 4 °C for 18 h. After three washes with 1 mL of PBS, FITC-conjugated His-Tag-specific antibody diluted to 1:200 in PB1% was added and incubated at room temperature for 1 h under protection from direct light. Glass slides were washed with 1 mL of PBS three times and then removed from the plate and sealed with SlowFade Diamond reagent (Thermo Fisher Scientific, United States). The reactions were visualized on a fluorescence microscope with a green filter (emission and excitation wavelengths of 482 nm and 532 nm, respectively) using an EVOS FLoid Imaging System (Thermo Fisher Scientific, United States) at ×10 magnification.

## Results

### Expression of TIM-3-ECD in *E. coli* BL21 (DE3)

**FIGURE 1:**
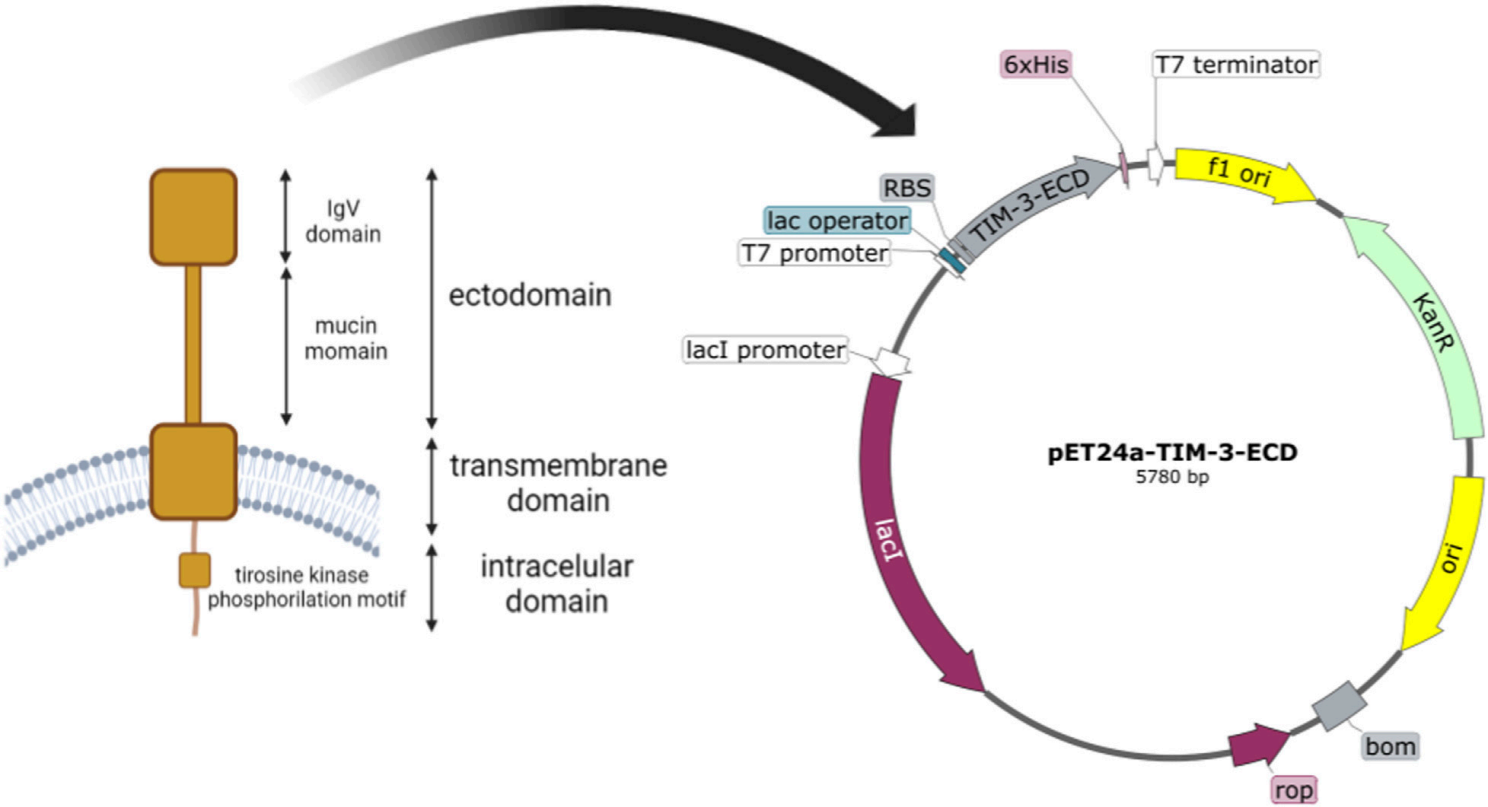
Schematic representation of TIM-3 native structure and construct map of pET24a-TIM-3-ECD. The figure depicts membrane-anchored TIM-3 and its structural domains, with the ectodomain highlighted by a red square. The *E. coli* optimized coding sequence of the TIM-3 ectodomain was then cloned into the pET-24-a expression vector. T7 Promoter: the T7 promoter region responsible for transcription initiation. Ribosome binding site (RBS): the sequence that facilitates the binding of ribosomes for translation initiation. TIM-3-ECD: the coding sequence for the ectodomain of the TIM-3 protein. 6xHisTag: a polyhistidine tag fused to the C-terminus of TIM-3-ECD for purification. Kanamycin resistance gene (KanR): a selectable marker conferring resistance to kanamycin. Origin of replication (ori): a sequence that enables the replication of the plasmid in host cells.

The TIM-3 protein structure consists of 21 amino acid molecules of a signal peptide, 181 amino acid molecules of the extracellular domain (ECD), 21 amino acids of transmembrane moieties (TM), and 78 amino acids of the cytoplasmic domain (CDM). The DNA fragment encoding TIM-3-ECD (181aa, 21 kDa) was synthesized into the kanamycin-resistant expression vector pET-24a *in-frame* with ATG initiation codon and poli-histidine tag (6xHis) coding sequence, resulting in the construction of the recombinant pET-24a-TIM-3-ECD vector, as shown in Figure 1. The primary structure characteristic of the recombinant protein was analyzed using the ProtParam tool (Expasy) and predicted to be a 190-residue protein (which comprises the 181 amino acids from TIM-3-ECD in addition to 6xHistag and restriction site amino acid residues), with 21,237.09 Da. The recombinant TIM-3-ECD isoelectric point was evaluated to be approximately 6.64, providing relevant information about the suitable pH for reaction buffers. Finally, the recombinant TIM-3-ECD instability index (II) was computed to be 28.86, classifying the protein as stable.

**FIGURE 2:**
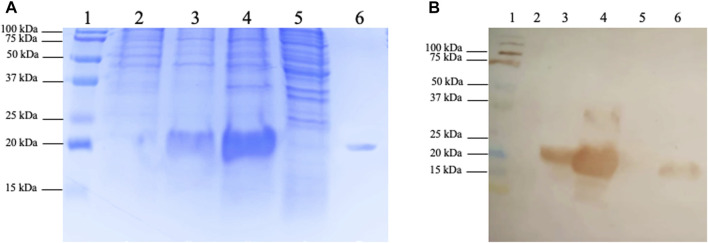
SDS-PAGE and immunodetection of recombinant TIM-3-ECD expression and recovery from inclusion bodies. **(A)** SDS-PAGE; lane 1, kaleidoscope (BioRad) molecular weight marker; lane 2, non-induced bacteria lysate fraction; lane 3, induced bacteria lysate fraction; lane 4, lysis insoluble fraction; lane 5, lysis soluble fraction; lane 6, inclusion bodies solubilized protein recovery fraction. **(B)** Immunodetection on a nitrocellulose membrane using 6xHisTag specific antibody; lane 1, kaleidoscope (BioRad) molecular weight marker; lane 2, non-induced bacteria lysate fraction; lane 3, induced bacteria lysate fraction; lane 4, lysis insoluble fraction; lane 5, lysis soluble fraction; lane 6, inclusion bodies solubilized protein recovery fraction.

Electrophoresis analysis demonstrated that the recombinant protein was present entirely in the insoluble fraction of induced bacterial lysis, with no detectable trace of the protein in the soluble fraction (Figure 2) even after several attempts to induce soluble cytoplasmic expression by changing the induction IPTG concentration and culture temperature (data not shown). The first attempt to obtain biologically active protein from inclusion bodies was by traditional denaturation methods, using 8 M urea to solubilize the inclusion bodies followed by recombinant protein refolding using different methodologies. Unfortunately, whole-produced recombinant protein was lost because of precipitation after this process (data not shown). To recover the TIM-3-ECD as a soluble protein from the inclusion bodies, an alternative solubilization process strategy was employed, avoiding harsh denaturation processes where inclusion body solubilization techniques did not result in recovery. All protein yield precipitated after refolding (data not shown). Figure 2A shows a ∼20 kDa single band on the solubilized fraction (Figure 2A, lane 6), indicating the absence of contaminant bands in solubilized samples, which suggests high purity despite the absence of purification steps. Recombinant protein production was confirmed by immunodetection with a 6xHis-specific antibody (Figure 2B). Due to the high amounts of recombinant protein produced by the pET expression system under strong induction conditions (up to one-third of the total protein mass of the cell in some cases), it is reasonable to suggest that TIM-3-ECD was the majority protein present at the inclusion bodies, explaining the absence of visible contaminant bands in solubilized sample (Figure 2A, lane 6). This solubilized fraction, which showed a final yield of 90 mg/L, was used to further biochemically characterize the recombinant TIM-3-ECD protein, in addition to its use in functional assays to validate the recombinant protein.

#### TIM-3-ECD biochemical characterization

**FIGURE 3:**
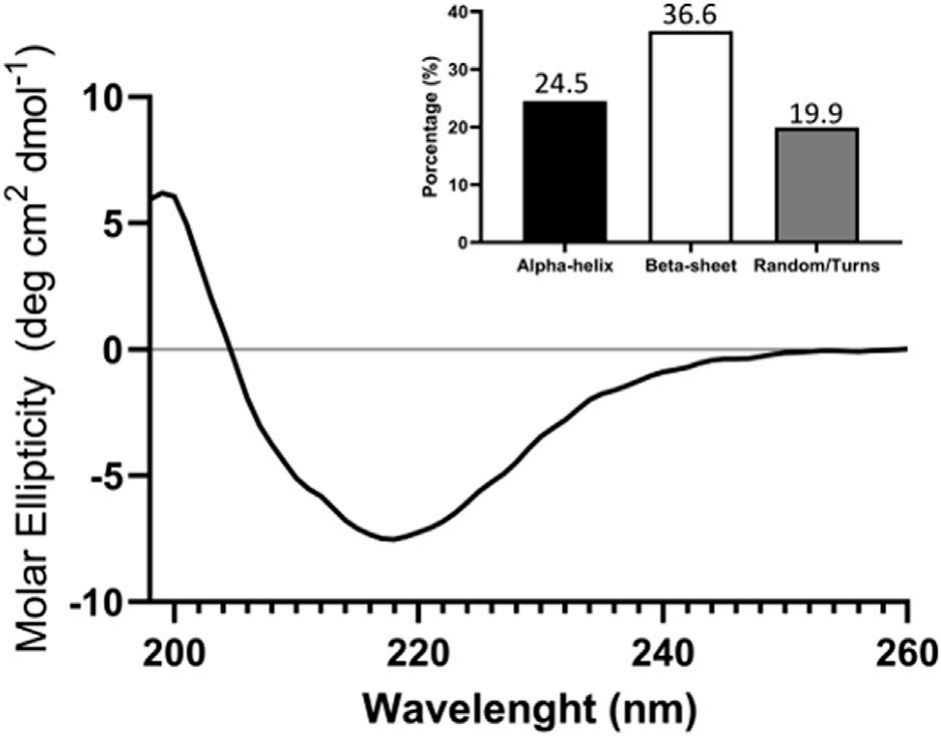
circular dichroism (CD) analysis of recombinant TIM-3-ECD. Analysis was performed between 190 and 260 nm. The column graph displays the proportional contribution of each secondary structure formation for global protein secondary structure as a result of convolutional analysis of CD collected data. The assay was performed in duplicate with two different recombinant protein batches.

The first step in characterizing the obtained TIM-3-ECD was to evaluate if the recombinant protein was able to retain its correct structure. By circular dichroism (CD) spectroscopy it was possible to analyze the secondary structure of TIM-3-ECD. As shown in Figure 3, the CD spectrum showed a characteristic positive peak at 200 nm and a negative peak at 220 nm, indicating the presence of beta-strand structures in the protein. Based on CD spectrum analysis, the secondary structure content of TIM-3-ECD was estimated to be approximately 36.6% alpha-helix, 24.5% beta-sheet, and 19.9% random coil (Figure 3). This result conformed with the presence of the native TIM-3-ECD IgV-like domain, predominantly formed by the beta-strand structure, indicating that the recombinant ectodomain had similar structural features to its native conformation.

**FIGURE 4:**
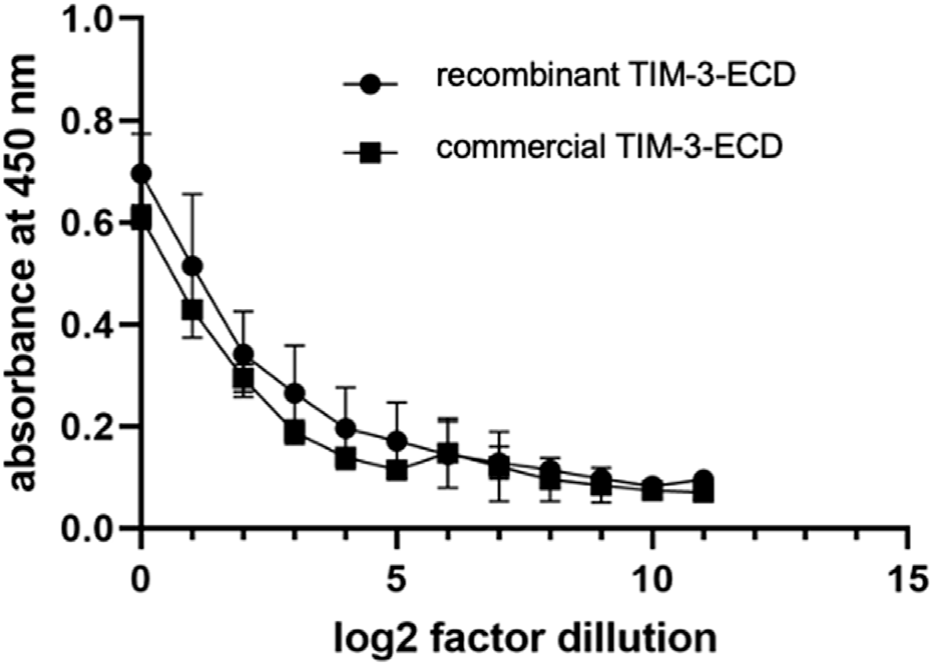
Indirect immunoenzymatic assay (ELISA) performed with the soluble recombinant ectodomain of TIM-3 obtained from the solubilization of inclusion bodies (circle) and from expression in HEK-293 human cell line (square). The immobilized protein was serially diluted in log2 from an initial concentration of 10 μg/mL. Values represent mean absorbance at 450 nm of three independent experiments. Error bars represent the standard deviation of three independent experiments.

Indirect ELISA was performed to evaluate if the recombinant TIM-3-ECD has the same immunoreactivity with a structure-dependent anti-TIM-3 antibody, as compared with commercial TIM-3-ECD produced in mammal cells. Figure 4 shows that the antibody specifically recognized the recombinant TIM-3-ECD, indicating its immunoreactivity. The antibody binding was dose-dependent and similar to commercial TIM-3-ECD, which indicated that the molecule’s structural properties were retained after recovery from inclusion bodies. The ELISA assay was successfully repeated several times during a 6-month period, which suggests that the recombinant protein maintains its structural stability; moreover, during this time the sample did not show any aggregation signal (data not shown). These results were encouraging for further investigating the recombinant molecule’s activity.

### TIM-3-ECD functional characterization

Finally, functional validation of TIM-3-ECD was performed, assessing the ability of the recombinant protein to interact with native ligands in their native state. Immune cell activation and immunofluorescence assays validated the interaction of recombinant TIM-3-ECD with primary immune cells and with lung tumor cells, respectively.

**FIGURE 5:**
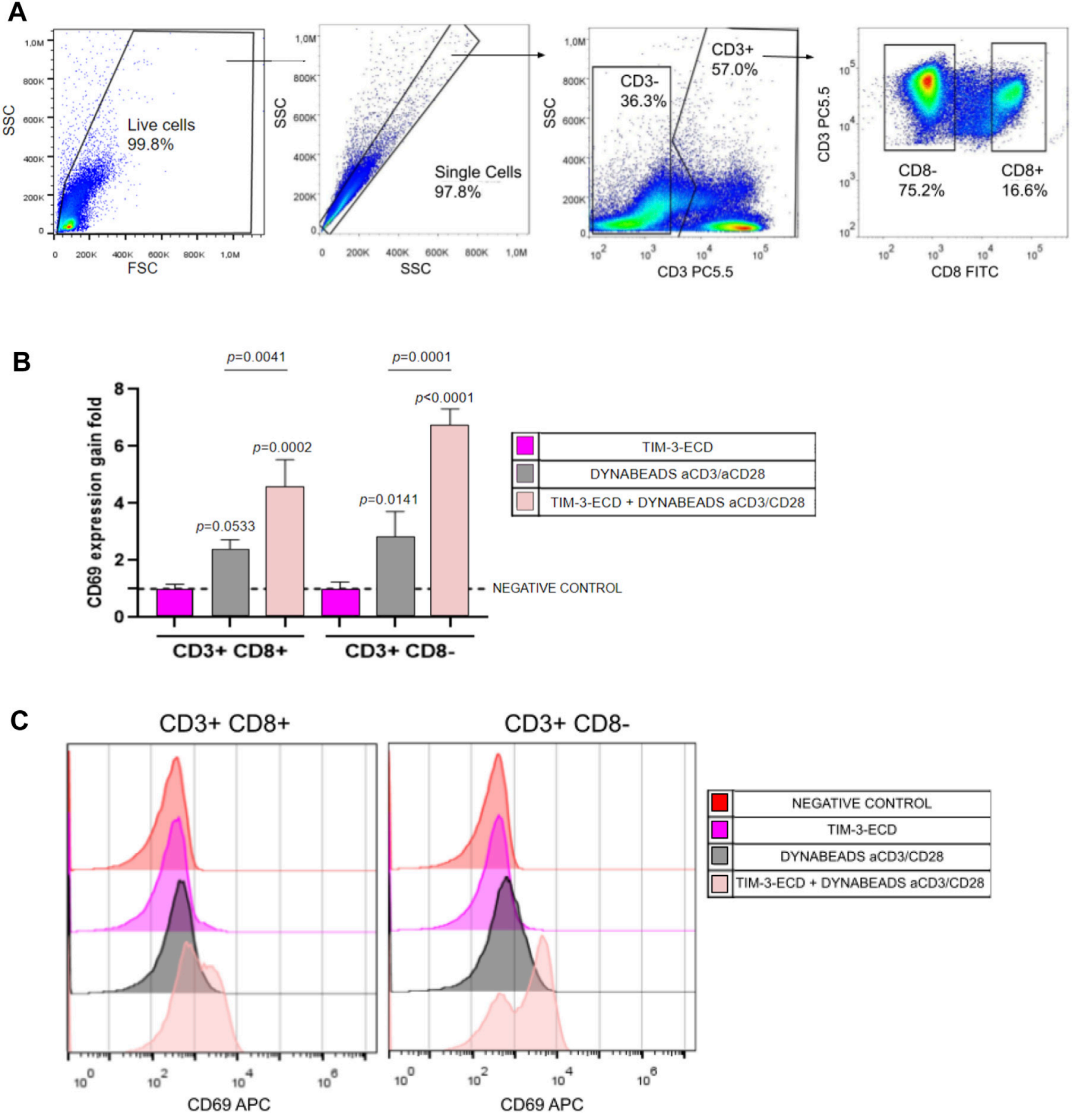
Flow cytometry analysis of immune activation assay with TIM-3-ECD. **(A)** Gate analysis. Cells were gated in CD3 and further gated on CD8^+^ or CD8^−^. Moreover, the CD69 activation marker was analyzed on CD3^+^CD8^+^ and CD3^+^CD8^−^ T cells. **(B)** CD69 gain expression analysis. Values represent the gain fold of the median fluorescence signal of the CD69 signal relative to the negative control without incubation with TIM-3-ECD and Dynabeads™ Human T-Activator CD3/CD28 (Thermo Fisher Scientific, United States). Error bars represent the standard deviation of three independent experiments. **(C)** Representative histogram of CD69 expression analyzed in all groups. Groups were analyzed in a one-way ANOVA test, with *p* values above bars representing the statistical significance relative to the negative control. The assay was performed in three independent experiments.

Flow cytometry analysis of human PBMCs provided important insights into the T cell activation relationship and modulation by recombinant TIM-3-ECD. Flow cytometry analysis was initially performed on CD3^+^ gated cells and, further, on CD8^+^ and CD8neg T cells. PBMCs pre-incubated with recombinant TIM-3-ECD led to an increase in the expression of the stimulation marker CD69 on both CD3^+^CD8^+^ and CD3^+^CD8neg T cells stimulated by Dynabeads™ Human T-Activator CD3/CD28 (Thermo Fisher Scientific, United States) (Figure 5). Recombinant TIM-3-ECD provides an additive effect of CD69 upregulation on activated T cells and may interact with specific ligands in these cells. Activation by recombinant TIM-3-ECD may extend to other populations of T lymphocytes, such as NK cells, which are CD3^−^ and were not evaluated here. Further investigation may reveal more details about the mechanism of recombinant TIM-3-ECD immune modulation in *in vitro* assays, contributing to its function characterization and providing insights about its potential therapeutic applications as a tool to further study TIM-3 ligands. Moreover, incubation of immune cells with recombinant TIM-3-ECD alone did not induce activation signaling in PBMCs, excluding the influence of potential agents such as detergent and/or bacterial endotoxins (e.g., LPS) in the preparation of recombinant TIM-3-ECD samples.

**FIGURE 6:**
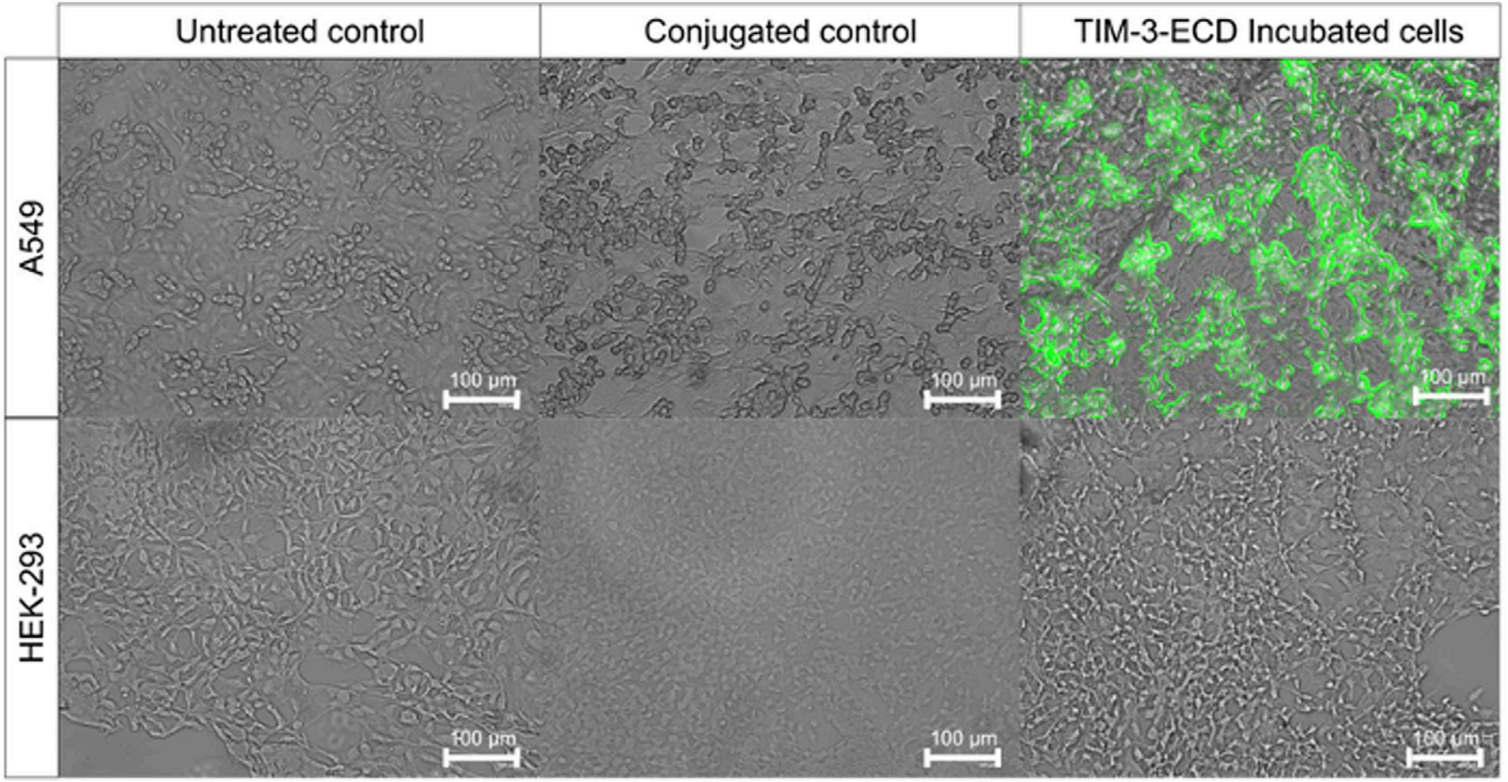
Immunofluorescence assay of recombinant TIM-3-ECD incubated with A549 and HEK-293 cell lines. Cells were incubated for 1 h with recombinant TIM-3-ECD, fixed with paraformaldehyde 4% (v/v), and labeled with anti-6xHis-specific FITC antibody. Controls are represented by non-labeled (untreated) and anti-6xHis labeled (conjugated) cells, without previous TIM-3-ECD incubation. Cells were visualized on a fluorescence microscope with a green filter (emission and excitation wavelengths of 482 nm and 532 nm, respectively) using an EVOS FLoid Imaging System (Thermo Fisher Scientific, United States) with ×10 magnification.

Immunofluorescence analysis investigated the specific interaction between recombinant TIM-3-ECD and TIM-3 ligands expressed by the lung cancer A549 cell line. The HEK-293 cell line was used as a negative control since it has no known expression of TIM-3 ligand. The assay with cells incubated with recombinant TIM-3-ECD for 1 h revealed that the recombinant protein was located on the periphery of A549 cells, as evidenced by the location of the green fluorescence signal of the anti-6xHisTag specific FITC antibody on the cell membrane. Moreover, the HEK-293 cell line presented an absence of green fluorescence signal, suggesting that the recombinant TIM-3-ECD was able to interact specifically with A549 membrane proteins. This cell is known to express CEACAM-1, which is believed to be a TIM-3 ligand (Figure 6) (Anderson and Anderson, 2006). However, further analysis must be performed to confirm this hypothesis.

Together, these results suggest that the established protocol of immune checkpoint ectodomain production in bacterial cytoplasm and refolding from inclusion bodies can produce structurally a stable and active recombinant TIM-3-ECD molecule, able to interact with and trigger in some way immune cell stimulation. However, further investigation must be done to establish the nature of these interactions and activations.

## Discussion

The advent of molecular biology has made it possible to use heterologous hosts for the protein and protein complex overexpression. Highly purified and well-characterized recombinant protein production has become a major task for the protein chemist within the pharmaceutical industry, and a tremendous wealth of knowledge has been accumulated on the subject as well as on using the enterobacterium *Escherichia coli* for this proposal (Terpe, 2006; Fernández and Vega, 2016). The main advantages of *E. coli* for protein production are well established: it has extremely fast growth kinetics, can achieve high cell densities, culture media and reagents are inexpensive, it has well-characterized genetics, and transformation with expression constructs is straightforward, with many options available for cloning vectors and mutant host strains (Fernández and Vega, 2016). On the other hand, despite its advantages, *E. coli* has certain limitations, such as inclusion bodies formation, LPS contamination, incomplete synthesis, degradation by proteases, and the lack of post-translational modifications (Hattab et al., 2014; Fernández and Vega, 2016). When it comes to membrane or disulfide bond-containing proteins, the situation is even more complex because they are difficult not only to produce but also to maintain in an active state in solution (Hattab et al., 2014).

Here, we aimed to present a feasible alternative protocol to obtain a recombinant version of the TIM-3 ectodomain as a biologically functional and stable molecule in the bacterial system. The TIM-3 ectodomain is a 21 kDa protein with 2 glycosylation sites (which will remain non-glycosylated once bacteria cannot perform post-translational modification) and 3 disulfide bonds, which are a challenge for recombinant protein stability and folding in cytoplasmic bacterial systems (Terpe, 2006).

Our study established, for the first time, an efficient method of biologically active TIM-3 ectodomain production by solubilizing the protein expressed as inclusion bodies in the *E. coli* cytoplasmic compartment. This method relied on the application of high hydrostatic pressure to solubilize inclusion bodies, with relatively mild conditions used for solubilizing aggregated proteins when compared to methods that make use of high concentrations of denaturing agents (Morganti and Chura-Chambi, 2023). Indeed, we attempted to use a traditional desaturating protocol (using 8 M urea) followed by refolding (using several protocols), with no success on the recovery of structured and functional protein. On the other hand, the application of high hydrostatic pressure has previously been determined to be an efficient method of recovering proteins from inclusion bodies (Chura-Chambi et al., 2018; Chura-Chambi et al., 2022). This soft solubilization method aimed to preserve the existing protein structures, possibly preserving the structure as the native-like conformation that recombinant TIM-3-ECD displayed in the structural analyses performed in this work.

Previous work involving the recombinant expression of immune checkpoint ectodomains, such as PD-1, PD-L1, and CTLA4 in bacteria, also used inclusion bodies produced in *E. coli* to obtain biologically active proteins (Cox et al., 1999; Li et al., 2004; Xu et al., 2006; Kalim et al., 2017; Mohammadzadeh et al., 2019; Zhansaya et al., 2020; Zhansaya et al., 2022). Despite the success of obtaining the IgV domain of the ectodomain of TIM-3 from bacteria in previous work (Cao, et al., 2007), this is the first study that obtained a complete and active TIM-3 ectodomain from this expression platform.

The TIM-3-ECD obtained in this work was able to retain at least part of its secondary structure, as shown by circular dichroism (Figure 3), and the non-glycosylated monomer was able to interact with immune cells and the A549 cell line, as well as displaying increased expression of the stimulation marker CD69 in activated CD3^+^ cells (Figure 5), even in the absence of post-translational modifications, suggesting that there must be some key N-linked glycosylation independent interaction occurring.

Despite having an identical primary structure to the soluble form of native TIM-3 or sTIM-3 (Clayton et al., 2015), the absence of interactions mediated by post-translational modifications may underlie the differentiated behavior of immune cells in the functional assay of incubation with bacterially produced TIM-3-ECD. It was previously shown that TIM-3 expression on dendritic cells may negatively regulate their function, impacting their production of IL-12 and tumor control (Maurya et al., 2014; Gujar et al., 2016). Although sTIM-3 is associated with negative regulation of immune system cells, we show here that TIM-3-ECD was able to induce an additive activation effect on bead-stimulated T cells *in vitro* (Figure 5). This contrast may indicate that the ectodomain of TIM-3 obtained in this work establishes a different network of interactions than its soluble form, directing immune cell activity toward stimulation. Future studies should demonstrate the ability of TIM-3-ECD to neutralize the interaction between TIM-3 and its ligands, through competition assays that may characterize the molecule as a TIM-3 antagonist. In addition, considering that our assays were performed in total PBMCs, we need to address in future studies which immune cell types may participate in this activation phenomenon.

Several reports described the interactions between TIM-3 and ligands such as Galactin-9 (Gal-9), carcinoembryonic antigen-related cell adhesion molecule 1 (CEACAM-1), and phosphatidylserine as being responsible for cell signal triggering and its immune modulation role (Anderson and Anderson, 2006; Boenisch et al., 2010; Acharya et al., 2020). Others specifically suggest that TIM-3 N-linked glycosylation regions interact with Galactin-9 and trigger the regulation signaling pathways; however, there is no consensus regarding which molecule is interacting with this region. Previous findings reveal that Gal-9 neutralizing is not a requirement for the functionality of TIM-3 antagonizing antibodies (Cao, et al., 2007; Anderson, 2012; Leitner et al., 2013; Gonçalves Silva et al., 2017; Sabatos-Peyton et al., 2018). Furthermore, other IgV regions were described to interact with CEACAM-1 and phosphatidylserine; however, recently, De Sousa Linhares et al. (2020) showed that TIM-3 functions are independent of CEACAM-1. The A549 cell lines are known to highly express the membrane receptor CEACAM-1, and interaction with this protein would reinforce previous findings that suggest that the binding of TIM-3 with its ligands utilizes interaction sites unrelated to the galectin-9 binding site (Cao et al., 2007). However, given the controversy regarding the interaction between CEACAM-1 and TIM-3 (Singer et al., 2010; Vitenshtein et al., 2016; De Sousa Linhares et al., 2020), a more specific analysis should be performed to infer that the recombinant TIM-3-ECD and cell surface interaction shown here is the result of the interaction with CEACAM-1 or any other unknown ligand.

It is noteworthy that the methodology for recombinant TIM-3 ectodomain recovery from inclusion bodies produced by bacteria proved to be a feasible way to obtain a biologically active extracellular domain of an immune cellular receptor. As many immune cellular receptor ectodomains share structural properties with TIM-3-ECD, such as disulfide bonds and glycosylation sites (Acúrcio et al., 2018), it may be viable to extend the methods established in this work as an alternative way to obtain relevant novel active immune proteins in the low-cost bacterial expression platform (de Marco et al., 2019; Slouka et al., 2019). The well-established industrial utilization of microbial factories such as *E. coli* to produce biopharmaceuticals represents a great opportunity to overcome the major bottlenecks faced by biopharmaceutical production, currently mostly performed in mammalian cells (Berlec and Strukelj, 2013).

Moreover, the results suggest that TIM-3-ECD produced in bacteria may represent an effective TIM-3 competitor in therapeutic applications and a suitable antigen for anti-TIM-3-specific antibody selection for immunotherapy. However, a further evaluation of this molecule’s interactions, comparing it with native or available TIM-3-ECD from mammal cells, must be performed to confirm this potential. Not least importantly, this recombinant molecule, because it lacks glycosylation, may be a useful tool to further study the TIM-3 ligands’ immune modulation N-glycosylation independent role.

## Data Availability

The datasets presented in this study can be found in online repositories. The names of the repository/repositories and accession number(s) can be found in the article/Supplementary material. The URL of the paper dataset publication on Butantan Institute repository https://repositorio.butantan.gov.br/handle/butantan/4918.
